# Supplementary material for: Collaborative intelligence in AI: Evaluating the performance of a council of AIs on the USMLE
Source: PLOS Digit Health. 2025 Oct 9;4(10):e0000787. doi: 10.1371/journal.pdig.0000787 (PMC12510544; doi:10.1371/journal.pdig.0000787)
Supplement: S2 File — (ZIP) [file pdig.0000787.s002.zip › S2 Data Raw data files containing unprocessed question inputs and LLM outputs including transcripts of deliberations/STEP3_Question-116-1_Type-MC-NJ.docx]

**Facilitator** **[Round 1]:** A 12-year-old girl with type 1 diabetes mellitus is brought to the emergency department by her parents because of a 2-day history of nausea, vomiting, and decreased oral intake. Medications are insulin glargine and insulin aspart. She decreased her insulin dose at the onset of symptoms but her condition has since worsened and she now is unable to consume solids or liquids without subsequent vomiting. She reports feeling dizzy on standing. Medical history is otherwise unremarkable. She is at the 50th percentile for height, weight, and BMI. Vital signs are temperature 37.8°C (100.1°F), pulse 118/min, respirations 27/min, and blood pressure 85/47 mm Hg. Pulse oximetry on room air shows an oxygen saturation of 99%. Physical examination discloses dry mucous membranes, delayed capillary refill time, and poor skin turgor. Palpation of the abdomen discloses diffuse tenderness with no rebound or guarding. The remainder of the physical examination discloses no abnormalities. Results of laboratory studies obtained on arrival are shown: Serum Urea nitrogen - 28 mg/dL Creatinine - 1.2 mg/dL Na⁺ - 126 mEq/L K⁺ - 4.3 mEq/L Cl⁻ - 95 mEq/L HCO3⁻ - 9 mEq/L Glucose - 563 mg/dL Arterial blood gas values on room air HCO3⁻ 10 mEq/L Partial Pressure of Oxygen - 98 mm Hg Partial Pressure of Carbon Dioxide - 22 mm Hg pH - 7.20 Boluses of intravenous 0.9% saline are administered and an infusion of insulin is initiated. Three hours later the patient becomes somnolent and develops a headache. She has had one episode of emesis and one episode of incontinence. Her urine output has been 30 mL/h. Results of follow-up serum laboratory studies are shown: Urea nitrogen - 24 mg/dL Creatinine - 1.1 mg/dL Na⁺ - 127 mEq/L K⁺ - 3.7 mEq/L Cl⁻ - 98 mEq/L HCO3⁻ - 11 mEq/L Glucose - 287 mg/dL Which of the following is the most appropriate intravenous pharmacotherapy? (A) Bicarbonate (B) Dexamethasone (C) 50% Dextrose (D) Furosemide (E) Mannitol

**AI_E:** RESTATING THE QUESTION: The question revolves around a 12-year-old girl diagnosed with type 1 diabetes mellitus who initially presented with nausea, vomiting, decreased oral intake, and dizziness upon standing, which later progressed into somnolence and headache. Laboratory values indicate high serum glucose and altered levels of electrolytes and blood gases, possibly indicative of multiple factors: insulin deficiency, dehydration, and ketoacidosis. We were asked to determine which intravenous pharmacotherapy would be most appropriate for her condition given the options: Bicarbonate, Dexamethasone, 50% Dextrose, Furosemide, and Mannitol. PLANNING: The high serum glucose and the girl's medical history point towards inadequate insulin signaling, which explains her symptoms as classic signs of diabetic ketoacidosis (DKA). The decrease in insulin dosage could have led to inadequate glucose intake by her cells, thereby triggering fat metabolism, and consequently, ketone body production. The symptoms and evident of DKA are clear: nausea, vomiting, loss of fluids (dehydration), decreased oral intake, and acidic blood (indicated by a reduced pH and bicarbonate level). Managing DKA requires restoring the patient's hydration status, correcting electrolyte imbalances, and reinstating effective insulin signaling. CLINICAL REASONING: Given the laboratory results, it seems the girl's hydration has been somewhat improved, as seen by the slight decrease in urea nitrogen and creatinine alongside an uptick in urine output. Improving hydration is vital in such scenarios as it aids in flushing out acids and helps reduce blood viscosity levels. Next, there's the issue of electrolyte imbalance. Most noticeable was the slight increase in sodium level; something commonly seen in DKA treatment as glucose and ketone bodies are flushed out, and the body compensates by retaining sodium. CONCLUDING: Looking at the following step, insulin administration has commenced, and glucose levels have dropped significantly, suggesting a response to insulin therapy. Despite these relative improvements, the patient developed headache, somnolence, and has experienced an episode of emesis and incontinence. These symptoms could be suggestive of cerebral edema, a severe and often fatal complication of DKA management in children. This complication arises primarily due to rapid correction of glucose and osmolality in the blood, causing water to move into the brain tissue, leading to swelling. RESPONSE: Given this clinical picture, the most appropriate intravenous pharmacotherapy would be Mannitol (option E) -- an osmotic diuretic. It works by increasing serum osmolality to prevent water movement into the brain tissue, therefore reducing cerebral edema. Early detection and treatment are crucial, as cerebral edema can swiftly lead to irreversible brain damage or death.

**AI_C:** RESTATING THE QUESTION: A 12-year-old female patient with type 1 diabetes, currently on insulin therapy, presents with symptoms indicative of diabetic ketoacidosis (indicated by high glucose and low bicarbonate levels). After treatment with saline and insulin, her condition seems to worsen, with symptoms of cerebral edema (headache, emesis, incontinence, and somnolence) developing. Given these conditions and the lab results provided, we are tasked to identify the most suitable intravenous pharmacotherapy. PLANNING: The assumption here is that we need to address both the dehydration caused by the initial diabetic ketoacidosis (DKA) and the resulting cerebral edema. Physiologically, high blood glucose levels (hyperglycemia) during DKA lead to high levels of ketones causing osmotic diuresis, electrolyte imbalance, severe dehydration, and potentially to the cerebral oedema if not treated properly. CLINICAL REASONING: Initial lab results and clinical presentations suggest a hyperglycemic state. Her high glucose, low bicarbonate, and lowered pH are indicative of DKA. She is treated with fluid resuscitation (0.9% saline) and insulin, bearing in mind her cerebral oedema signs like a headache and drowsiness. CONCLUDING: On close examination, the glucose levels drop but do not normalize entirely, and the bicarbonate levels slightly improve. However, cognitive symptoms seem to worsen, with the patient developing a headache and somnolence, indications of cerebral edema. A re-evaluation of her pharmacotherapy is needed to address these complications. RESPONSE: Based on her clinical manifestation after primary treatment, the most appropriate next step will be to administer Mannitol (E), an osmotic diuretic used to decrease cerebral edema by creating an osmotic gradient that draws fluid out of the swollen brain tissues. Patients with DKA are vulnerable to cerebral oedema, particularly during the early stages of treatment. The syndrome is erroneously attributed solely to a rapid decrease in plasma osmolality induced by treatment, but it often happens before plasma glucose levels have substantially fallen, implicating other factors (e.g., changes in effective osmoles such as β-hydroxybutyrate) in its development. It is crucial to recognize high-risk patients and to initiate timely, appropriate treatments. Other options like Bicarbonate, Dexamethasone, 50% Dextrose, or Furosemide would not be the first line of treatment for cerebral edema in this case.

**AI_A:** RESTATING THE QUESTION: The case presented here introduces a 12-year-old girl with type 1 diabetes mellitus who has been suffering from illness characterized by nausea, vomiting, and decreased oral intake over the past two days. She is unable to consume solids or liquids, and is presenting signs of dehydration. Initial lab studies reveal a high glucose level and abnormal serum parameters, indicative of diabetic ketoacidosis (a serious diabetes complication). After administration of saline and insulin, her symptoms become more severe with an instance of emesis, incontinence, decreased consciousness, and a headache. A follow-up serum lab study shows an improvement in her glucose level and renal function, but her serum bicarbonate remains below normal, indicating persisting metabolic acidosis. Amidst this clinical scenario, we are tasked with identifying the most suitable intravenous pharmacotherapy. PLANNING: Our central assumption is that the intravenous pharmacotherapy must address the girl's diabetic ketoacidosis (DKA), and more specifically, the persisting metabolic acidosis. It's plausible to question this assumption: could her somnolence and headache be due to cerebral edema, a complication of DKA, which requires a different therapeutic approach such as hyperosmolar therapy as with mannitol or furosemide? CLINICAL REASONING: In cases of DKA, initial fluid resuscitation with normal saline followed by an insulin regimen is the primary treatment to restore hydration and reverse the metabolic derangements. The persisting low serum bicarbonate and low pH from both initial and follow-up labs indicates ongoing metabolic acidosis despite treatment. The presentation of headache, somnolence and decreased level of consciousness might suggest cerebral edema, a complication of DKA especially in children, but it's also important to note that the child's renal function has progressively improved, evidenced by decreasing levels of urea nitrogen and creatinine. This points away from the use of furosemide or mannitol which are used to manage fluid overload or cerebral edema respectively; it also doesn't explain the ongoing metabolic acidosis. CONCLUDING: The girl's metabolic acidosis should be the priority. While it's expected to correct with ongoing insulin treatment and hydration, the persistently low bicarbonate level coupled with her worsened neurological symptoms may justify a temporary targeted treatment to normalize her acid-base balance as insulin and fluid therapy continue. RESPONSE: The most appropriate intravenous pharmacotherapy in this case would be (A) Bicarbonate.

**AI_B:** RESTATING THE QUESTION: A young patient diagnosed with type 1 diabetes experiencing hyperglycemia, confusion, headache, vomiting, and incontinence, among other symptoms has undergone initial treatment that has included the administration of saline and insulin. However, her symptoms continue, leading to the question of which intravenous pharmacotherapy would be most appropriate next. The options are bicarbonate, dexamethasone, 50% dextrose, furosemide, and mannitol. PLANNING: Given the clinical features suggesting Diabetic ketoacidosis (DKA) — hyperglycemia, acidemia, electrolyte imbalance (hyponatremia), and high anion gap metabolic acidosis — it's likely that we'd need a treatment that attends specifically to these factors. Bicarbonate is used for acidosis; Dexamethasone is a corticosteroid (which would raise blood glucose); 50% Dextrose would elevate blood glucose and is typically used in hypoglycemia, not hyperglycemia; Furosemide, a diuretic, would help reduce fluid volume but might exacerbate dehydration and Mannitol, an osmotic diuretic, is typically used for reducing cerebrospinal fluid pressure or intraocular pressure. CLINICAL REASONING: As the patient already has high blood glucose levels, treatments that would further raise this (Dexamethasone and 50% Dextrose) should be avoided here. Likewise, while Furosemide can help manage fluid levels, it could worsen the patient's dehydration, so this isn't an ideal choice. That leaves us with bicarbonate and mannitol. While bicarbonate could address the acidosis potentially associated with DKA, if the acidosis isn’t severe and there's already an insulin drip present, the body can naturally buffer the low pH. The important element to consider here is the patient's neurological status which has apparently worsened with somnolence, headache and one episode of incontinence — it could indicate cerebral edema, a potential complication of DKA treatment. Hence, the use of mannitol that decreases intracranial pressure might be more beneficial. CONCLUDING: While the patient has DKA evidenced by her initial laboratory findings (hyperglycemia, acidemia, hyponatremia, and a raised anion-gap metabolic acidosis), she has also exhibited signs of cerebral edema (somnolence, headache, incontinence). Bicarbonate therapy might help rectify the acidemia, but if the acidosis isn't too severe, the body can likely buffer this itself with insulin administration. Instead, addressing the potential cerebral edema, which can be a serious and life-threatening complication, becomes the priority. RESPONSE: Given these circumstances, the most appropriate intravenous pharmacotherapy is likely mannitol (E), which can help to decrease intracranial pressure and counter cerebral edema.

**AI_D:** (1) RESTATING THE QUESTION: The question is asking about the most suitable intravenous pharmacotherapy for a 12-year-old girl who has type 1 diabetes mellitus and is experiencing severe symptoms, including nausea, vomiting, impaired oral intake, dizziness, somnolence, a headache, and inconsistent urine output. Some of her laboratory findings suggest potential metabolic and fluid imbalances. (2) PLANNING: The basic assumptions here are that the girl's symptoms are directly related to her diabetes and that her condition could be a result of diabetic ketoacidosis (DKA), a potentially life-threatening complication of type 1 diabetes characterized by hyperglycemia, metabolic acidosis, and excessive production of ketones in the body. This can result from decreased insulin utilization due to inadequate insulin therapy. The features of her clinical presentation and lab results (increased pulse rate, decreased blood pressure, dry mucous membranes, delayed capillary refill time, poor skin turgor, elevated glucose, and low bicarbonate level) align with DKA. An alternate assumption is that the patient could be experiencing cerebral edema, a serious, potentially life-threatening complication of DKA that can occur particularly in pediatric patients during the treatment of DKA. The clinical features suggestive of this are the development of somnolence and headache, and the occurrence of emesis and incontinence. To determine which assumption is more clinically relevant, the state of the patient's condition following the rehydration and insulin therapy along with the development of new symptoms must be carefully evaluated. (3) CLINICAL REASONING: The girl's symptoms developed when she reduced her insulin dosage. Later, following fluid resuscitation and insulin therapy in the ED, her somnolence, headache, emesis, and incontinence all suggest that cerebral edema might have started, alarming signs of a substantial complication arising during DKA treatment. The process for cerebral edema in this context is not precisely understood but is thought to be associated with shifts in fluid and electrolytes, specifically, a rapid decrease in blood sugar can induce an osmotic fluid shift from the intravascular compartment into the brain leading to cerebral edema. This phenomenon could be intensified by the rapid intravenous rehydration which along with the underlying acidosis may trigger vasogenic edema by disrupting the blood-brain barrier and allowing fluid to enter cerebral tissue. (4) CONCLUDING: Given the girl's clinical presentation post-treatments and her follow-up labs indicating improved hydration (decreased BUN, increased creatinine, mildly increased sodium), as well as the resolution of the metabolic acidosis and hyperglycemia (increased HCO3- and decreased glucose), the assumption of cerebral edema secondary to DKA treatment warrants more attention and underlines the requirement for an agent that could reduce cerebral edema. (5) RESPONSE: In this situation, the most appropriate intravenous pharmacotherapy would be (E) Mannitol - an osmotic diuretic that can help decrease intracranial pressure by creating an osmotic gradient that draws fluid out from the brain into the bloodstream, thereby reducing the cerebral edema.

**Facilitator** **[Round 2]:** Summary: AI_A believes that the most suitable intravenous pharmacotherapy for the patient is bicarbonate (A), as it would address the persisting metabolic acidosis, which is the priority in this case. AI_B, AI_C, AI_D, and AI_E, however, argue that the patient's worsening neurological symptoms suggest cerebral edema, a serious complication of DKA treatment. They propose that mannitol (E), an osmotic diuretic that can decrease intracranial pressure and counter cerebral edema, would be the most appropriate treatment. Key Differences: The key difference in the council members' perspectives lies in their interpretation of the patient's symptoms and lab results. AI_A focuses on the persisting metabolic acidosis, arguing that bicarbonate would help normalize the patient's acid-base balance. In contrast, AI_B, AI_C, AI_D, and AI_E emphasize the potential cerebral edema, suggesting that mannitol would be more beneficial in reducing intracranial pressure and managing this complication. Question for Council Members: Given the patient's persisting metabolic acidosis and the potential risk of cerebral edema, how would you prioritize these complications in the treatment plan? Would a combined approach using both bicarbonate and mannitol be feasible and beneficial in this case?. Based on your preceding response, how would you answer the original question: [A 12-year-old girl with type 1 diabetes mellitus is brought to the emergency department by her parents because of a 2-day history of nausea, vomiting, and decreased oral intake. Medications are insulin glargine and insulin aspart. She decreased her insulin dose at the onset of symptoms but her condition has since worsened and she now is unable to consume solids or liquids without subsequent vomiting. She reports feeling dizzy on standing. Medical history is otherwise unremarkable. She is at the 50th percentile for height, weight, and BMI. Vital signs are temperature 37.8°C (100.1°F), pulse 118/min, respirations 27/min, and blood pressure 85/47 mm Hg. Pulse oximetry on room air shows an oxygen saturation of 99%. Physical examination discloses dry mucous membranes, delayed capillary refill time, and poor skin turgor. Palpation of the abdomen discloses diffuse tenderness with no rebound or guarding. The remainder of the physical examination discloses no abnormalities. Results of laboratory studies obtained on arrival are shown: Serum Urea nitrogen - 28 mg/dL Creatinine - 1.2 mg/dL Na⁺ - 126 mEq/L K⁺ - 4.3 mEq/L Cl⁻ - 95 mEq/L HCO3⁻ - 9 mEq/L Glucose - 563 mg/dL Arterial blood gas values on room air HCO3⁻ 10 mEq/L Partial Pressure of Oxygen - 98 mm Hg Partial Pressure of Carbon Dioxide - 22 mm Hg pH - 7.20 Boluses of intravenous 0.9% saline are administered and an infusion of insulin is initiated. Three hours later the patient becomes somnolent and develops a headache. She has had one episode of emesis and one episode of incontinence. Her urine output has been 30 mL/h. Results of follow-up serum laboratory studies are shown: Urea nitrogen - 24 mg/dL Creatinine - 1.1 mg/dL Na⁺ - 127 mEq/L K⁺ - 3.7 mEq/L Cl⁻ - 98 mEq/L HCO3⁻ - 11 mEq/L Glucose - 287 mg/dL Which of the following is the most appropriate intravenous pharmacotherapy? (A) Bicarbonate (B) Dexamethasone (C) 50% Dextrose (D) Furosemide (E) Mannitol]

**AI_B:** To answer this question, we need to understand the context of the patient's condition and the possible treatment methods. The 12-year old female patient, diagnosed with Type 1 diabetes, arrived in the medical facility with worsened symptoms like nausea, vomiting, incapability to consume food or drinks, and dizziness on standing. Her current medications are insulin glargine and insulin aspart. Laboratory test results indicated diabetic ketoacidosis (DKA) based on high glucose levels, a low bicarbonate level, and acidemia, and she was treated for the same. However, hours after treatment commencement, her condition deteriorated, with new symptoms such as sleepiness, headache, vomiting, and incontinence. The urine output increased, indicating adequate renal perfusion and function, and glucose levels showed an improvement. The primary care aim currently is to treat the acute complication of DKA, specifically cerebral edema, and prevent further complications. The choice of medication should be tailored to address the most impactful condition in the present clinical scenario. It's hypothesized that cerebral edema is a potential and critical consequence to consider in this context, as evidenced by the patient's somnolence, headache, emesis, and incontinence, even though the metabolic acidosis is not fully rectified yet. With this alternate assumption, it's important to note that cerebral edema in DKA is most likely due to a rapid decrease in extracellular osmolality following the treatment with insulin and fluids, which drives water into the brain cells. Intravenous mannitol can counteract these effects by increasing the serum osmolality and drawing fluid out both the swollen brain cells as well from the blood-brain barrier itself thereby restoring cerebral perfusion and mitigating the symptoms of cerebral edema such as altered mental status. Mannitol treatment needs to be prioritized as prompt intervention can be lifesaving considering the potential rapid progression and criticality of cerebral edema in DKA patients. Bicarbonate administration would help with metabolic acidosis, but it isn't the top priority in this scenario because bicarbonate levels are expected to improve gradually with the treatment of DKA itself. Also, routine bicarbonate administration in DKA is not recommended because it doesn’t change clinical outcomes and has potential side effects like paradoxial CNS acidosis and hypokalemia. Thus, based on my clinical reasoning, I'd advocate the administration of mannitol to deal with the highly probable cerebral edema this patient is experiencing. Mannitol's action as an osmotic diuretic can alleviate cerebral edema by decreasing intracranial pressure, and hence it needs to be prioritized over other treatments at this point. Therefore, my final answer to the question is (E) Mannitol.

**AI_A:** Restatement: We are inquiring about the most suitable intravenous medication for a 12-year-old girl who is a diagnosed case of type 1 diabetes mellitus and is currently brought to the emergency department with a 2-day history of nausea, vomiting, and decreased oral intake. She has been experiencing severe symptoms including dizziness and vomiting with any oral intake. Initial laboratory findings revealed markedly high glucose and a clear picture of metabolic acidosis. The patient has been given normal saline boluses and insulin. However, the condition has not improved substantially and she developed more alarming symptoms like a headache and somnolence, raising suspicion of cerebral edema, a complication that can occur with rapid correction of her situation. Updated laboratory findings did show a decrease in glucose levels and slight improvement in metabolic acidosis, yet the patient's condition continued to worsen. Plan: We need to consider two scenarios - the patient's persisting and severe metabolic acidosis, evident by her low blood HCO3⁻ levels, gives us an initial assumption to contemplate the use of bicarbonate. On the other hand, we need to consider the possibility of cerebral edema given her new neurological symptoms; this second assumption prompts us to contemplate the use of an osmotic diuretic like mannitol. It would be helpful to understand how we can rectify both complications simultaneously. Clinical Reasoning: Persisting metabolic acidosis despite insulin administration indicates that more needs to be done to balance the patient's system's acid-base status. Bicarbonate can directly counteract acid-base imbalances, proving beneficial for the patient's acidotic state. However, this approach neglects the patient's new neurological symptoms, which suggest the development of cerebral edema due to the rapid correction of hyperglycemia - a known risk in DKA treatment. Mannitol, an osmotic diuretic, draws water out of tissues (including the brain) into the blood, helping to reduce intracranial pressure and soher neurological symptoms. Conclusion: Balance is crucial in managing the acidosis and avoiding the potential onset of cerebral edema. Bicarbonate might address the immediate concern of ongoing metabolic acidosis. Still, it misses the severity of cerebral edema that can lead to grave consequences like herniation and death. On the other hand, mannitol might tackle potential cerebral edema but leaves the primary condition largely untouched. Hence, our approach should ideally consider both issues without overlooking one. Response: Given the severity of the symptoms, the most immediate concern is the suspected onset of cerebral edema indicated by her headache, somnolence, vomiting, and incontinence. Hence, starting the patient on Mannitol (E) would be the most appropriate immediate step to reduce her intracranial pressure potentially caused by cerebral edema in the setting of DKA treatment. This, however, doesn't negate the need to continue managing the patient's metabolic acidosis through continuing insulin and fluid management and reassessing the need for bicarbonate in the ongoing treatment plan. Making sure that the treatment is closely monitored to prevent any further complications is of utmost importance.

**AI_C:** RESTATING THE QUESTION: We are asked to identify the most effective intravenous pharmacological treatment for a 12-year-old girl with type 1 diabetes mellitus, who showed severe symptoms of diabetic ketoacidosis (DKA), such as hyperglycemia, metabolic acidosis, and signs of dehydration. DKA is a metabolic emergency that happens when there's not enough insulin leading to excessive production of ketone bodies that cause metabolic acidosis. She was given saline and insulin infusion as initial treatment. However, she later developed symptoms indicating possible cerebral edema, a severe and potentially life-threatening complication of DKA treatment seen primarily in pediatric patients, characterized by altered mental status and neurologic signs. PLANNING: The potential assumptions that could be drawn from the question include: (a) the girl's symptoms of DKA have resolved with the initial treatment, hence the elevated glucose levels and reduced bicarbonate levels are the priority for treatment; (b) the new symptoms the girl is experiencing are signs of cerebral edema, thus prioritizing this complication over the already improving metabolic derangements. Comparing these assumptions, the latter should be clinically prioritized because cerebral edema can be fatal if not treated immediately. CLINICAL REASONING: The girl's initial symptoms and laboratory results showed signs of DKA, presenting as hyperglycemia (glucose > 250 mg/dL), anion gap metabolic acidosis (low bicarbonate) and positive ketone bodies (not shown in data but can be assumed given her diagnosis). This necessitated treatment with fluid resuscitation (saline) to correct dehydration and insulin to suppress ketogenesis. Subsequently, she developed symptoms such as somnolence, headache, emesis, and incontinence, which are typically suggestive of cerebral edema. Such edemas occur as a complication in DKA treatment due to aggressive fluid resuscitation and rapid decrease in blood glucose causing a shift of water into the brain cells leading to swelling. CONCLUDING: Given the clinical scenario and following the assumption that the girl is likely experiencing cerebral edema, the treatment approach should thus focus on reducing brain edema to prevent further neurological deterioration. RESPONSE: Consequently, the most appropriate intravenous pharmacotherapy in this instance would be (E) Mannitol, an osmotic diuretic that helps decrease brain swelling by shifting water from the brain into blood vessels. The other options such as bicarbonate are not prioritized since the metabolic changes are already showing signs of improvement after initial treatment (glucose reduced to 287 mg/dL, bicarbonate slightly increased to 11 mEq/L). Moreover, treating the underlying cause (cerebral edema) will further help correct acidosis.

**AI_D:** RESTATING THE QUESTION: The patient, a 12-year-old girl with type 1 diabetes, presents with prolonged nausea, vomiting, dizziness, and inability to consume food or drinks without subsequent vomiting. She has decreased her insulin dose and has generalized abdominal tenderness. Venous blood tests reveal high blood glucose levels, low Bicarbonate (HCO3⁻) levels, and moderately decreased serum Sodium (Na⁺), indicating hyperglycemia, metabolic acidosis, and hyponatremia. Despite the provision of intravenous saline and insulin, her condition worsens, exhibiting neurological symptoms consistent with cerebral edemas such as headache, somnolence, vomiting, and incontinence. Her urinary output has also reduced. Following up lab tests show improvements in her blood glucose levels, BUN, serum creatinine, sodium, potassium, chloride, and bicarbonate levels. We need to determine the best intravenous pharmacotherapy among bicarbonate, dexamethasone, Dextrose 50%, furosemide, and mannitol, considering her symptoms and treatment progress. PLANNING: The key assumptions are: 1) ongoing metabolic acidosis requiring possible use of bicarbonate; 2) potential cerebral edema, therefore might need mannitol - an osmotic diuretic to reduce intracranial pressure; 3) insulin therapy causing hypoglycemia requiring possible use of Dextrose 50%; 4) excessive fluid accumulation justifying the use of furosemide; and 5) inflammatory condition that may benefit from dexamethasone. Evaluating these assumptions, we should prioritize potential cerebral edema due to its acuteness and fatal implications. CLINICAL REASONING: Diabetic ketoacidosis (DKA) in type 1 diabetic patients brings on hyperglycemia and metabolic acidosis due to low insulin, manifested by the patient's high glucose and low bicarbonate levels. The treatments provided, fluids, and insulin, rectify these issues as her updated lab results show. Yet, her worsening neurological conditions indicate possible cerebral edema--a rare but deadly complication of DKA treatment as it can lead to herniation and death. This rapid shifts of fluids may cause a dangerous drop in glucose and an osmotic effect leading to cerebral edema. Mannitol, an osmotic diuretic, can alleviate this by reducing intracranial pressure through extraction of water from the brain to the bloodstream, thus prioritizing its usage here. Bicarbonate, Dextrose 50%, Furosemide, and Dexamethasone, however, may not aptly address the current priority concern. CONCLUDING: While bicarbonate can help resolve the patient's lingering acidosis, other symptoms indicate a pressing issue – cerebral edema, one of the most severe complications in the treatment of DKA, which can be life-threatening if not promptly addressed. It is crucial to manage this condition effectively to prevent further neurological deterioration. RESPONSE: Therefore, the most appropriate intravenous pharmacotherapy for the given patient conditions would be Mannitol (Option E).

**AI_E:** 1) RESTATING THE QUESTION: The question involves a 12-year-old girl with Type 1 diabetes who was brought to the emergency department due to nausea, vomiting, and decreased oral intake for the past two days. With medication usage including insulin glargine and insulin aspart, she had decreased her insulin intake at the onset of symptoms, but her condition has since worsened to the point of not being able to consume solids or liquids without subsequent vomiting. Initial lab results show prominent features such as raised glucose levels and decreased bicarbonate levels. Later lab results after administering boluses of 0.9% saline and insulin infusion shows an improvement in glucose and some improvement in bicarbonate levels, but the girl develops signs of neurological impairment leading to confusion about the most appropriate intravenous pharmacotherapy between options (A) Bicarbonate, (B) Dexamethasone, (C) 50% Dextrose, (D) Furosemide, and (E) Mannitol. 2) PLANNING: The initial assumption relates to Diabetic Ketoacidosis(DKA) due to the observed high glucose levels, low bicarbonate levels (reflecting acidosis), and her known history of Type 1 diabetes and insulin usage. The symptoms like nausea, vomiting, inability to eat or drink relates to the insulin deficiency and volume depletion. However, from the observed symptoms such as somnolence, headache, emesis, and incontinence along with her lowered sodium levels, there may be an alternate assumption of complications arising such as cerebral edema. Therefore, our plan would be to judge which alternate assumption holds more weight considering the progress of her symptoms, prioritize the appropriate pharmacotherapy that would counter the most pressing issue and reason clinically based on evidenced medical knowledge. 3) CLINICAL REASONING: The symptoms noticed during the first visit such as dry mucous membranes, delayed capillary refill time, and poor skin turgor would clearly indicate a dehydration state. This along with corresponding lab results of raised glucose levels and and lack of bicarbonate(in line with the body compensating with accelerated breathing for acidosis) would guide us towards a decision of DKA. Subsequent treatment of IV saline and insulin did help in improving her glucose levels but she was now showing signs of cerebral complications(attested by her somnolence, headache after treatment). Decreasing sodium levels are a clear giveaway of hyponatremia leading to brain edema. Hence we have two directions to consider in this: Treatment for persisting DKA or treatment for cerebral edema. 4) CONCLUDING: The most appropriate choice of intravenous pharmacotherapy must be directly aimed at tackling the most pressing problem. Given that she is improving in terms of DKA symptoms as seen by her glucose levels coming under control and bicarbonate levels slightly raising, and there's emergence and progression of pressing new symptoms of headache, somnolence, vomiting, and incontinence, it is evident that brain edema is now the major concern. 5) RESPONSE: Therefore, considering the mentioned drug options in this case, the presence of cerebral edema should guide the choice of pharmacotherapy above the persisting DKA symptoms. Mannitol would be a more appropriate pharmacotherapy in this situation as it functions as an osmotic diuretic that would help in reducing the cerebral edema and associated symptoms with it. Hence option (E) Mannitol is the correct choice.

**Facilitator** The question pertains to a 12-year-old girl with type 1 diabetes mellitus who has been brought to the emergency department due to severe symptoms of diabetic ketoacidosis (DKA), including nausea, vomiting, and decreased oral intake. Despite initial treatment with saline and insulin, her condition has worsened, with new symptoms suggesting the onset of cerebral edema, a severe and potentially life-threatening complication of DKA treatment. The primary concern is to treat this acute complication and prevent further deterioration. Given the severity of the symptoms and the potential onset of cerebral edema, the most appropriate immediate step would be to start the patient on Mannitol, an osmotic diuretic that can help reduce intracranial pressure potentially caused by cerebral edema. Mannitol works by drawing water out of tissues, including the brain, into the blood, thereby helping to reduce intracranial pressure and alleviate neurological symptoms. This treatment should be prioritized due to the potential rapid progression and criticality of cerebral edema in DKA patients. However, this does not negate the need to continue managing the patient's metabolic acidosis through ongoing insulin and fluid management. Bicarbonate administration could help with metabolic acidosis, but it is not the top priority in this scenario as bicarbonate levels are expected to improve gradually with the treatment of DKA itself. Routine bicarbonate administration in DKA is not recommended due to potential side effects and lack of impact on clinical outcomes. Therefore, the consensus among the responses is that the most appropriate intravenous pharmacotherapy for this patient would be Mannitol (Option E). This approach should ideally consider both the ongoing metabolic acidosis and the potential onset of cerebral edema without overlooking one over the other. It is of utmost importance that the treatment is closely monitored to prevent any further complications.
